# Zoning of Integrated Quality Regions for *Alpinia officinarum* Hance Based on a Multi-Model Evaluation System

**DOI:** 10.3390/biology15040369

**Published:** 2026-02-22

**Authors:** Heng Jiang, Bin Huang, Tao Li, Ying Liu, Shuang Zhang, Quan Yang, Kunhua Wei

**Affiliations:** 1 Key Laboratory of State Administration of Traditional Chinese Medicine for Production & Development of Cantonese Medicinal Materials, Guangzhou Comprehensive Experimental Station of National Industrial Technology System for Chinese Materia Medica, Guangdong Engineering Research Center of Good Agricultural Practice & Comprehensive Development for Cantonese Medicinal Materials, School of Chinese Materia Medica, Guangdong Pharmaceutical University, Guangzhou 510006, China; jh15874556538@163.com (H.J.); 15362935395@163.com (T.L.); 19808032056@163.com (Y.L.); wenliang57321@163.com (S.Z.); 2 School of Pharmaceutical Sciences, Hunan University of Medicine, Huaihua 418000, China; huangbin@ynucm.edu.cn; 3 The First School of Clinical Medicine, Yunnan University of Chinese Medicine, Kunming 650500, China; 4 Yunfu Germplasm Resource Management Center, Yunfu 527300, China

**Keywords:** multi-model ensemble system, ArcGIS, *Alpinia officinarum* Hance, MaxEnt optimization, co-kriging interpolation, integrated quality regions

## Abstract

Understanding the spatiotemporal dynamics of medicinal plant distributions and their quality responses under climate change is essential for formulating forward-looking conservation and utilization strategies. In response to the increasing depletion of wild resources of *Alpinia officinarum* Hance, one of the ‘Ten Major Guangdong Medicinal Materials’, this study developed an integrated modeling platform incorporating nine algorithms. These included generalized linear models, machine learning techniques, and a MaxEnt model optimized using ENMeval (Regularization Multiplier (RM) = 3, Feature Class (FC) = LQH). The platform was applied to simulate habitat suitability evolution under current climatic conditions (1970–2000) and for two future periods (2050s: 2041–2060; 2090s: 2081–2100) across four Shared Socioeconomic Pathways (SSP126, SSP245, SSP370, and SSP585). Furthermore, Co-kriging interpolation was coupled to conduct a comprehensive quality zoning based on the dual “ecological-chemical” dimension. Analysis of key environmental factors revealed that the distribution of *A. officinarum* is primarily constrained by hydrothermal conditions, with a suitable annual temperature ranges from 19.96 to 29.05 °C and a dry-season precipitation requirement between 56.64 and 185.65 mm. Model projections indicate that future warming does not promote habitat expansion; instead, it drives a latitudinal shift in the suitability centroid toward lower latitudes. The cumulative effects of different emission pathways vary markedly: the high-emission scenario (SSP585) triggers severe habitat contraction by the 2090s, while habitat loss under the SSP370 scenario remains relatively manageable. By overlaying the spatially heterogeneous distribution of galangin, this study delineated southeastern Yunnan, southeastern Guangxi, southwestern Guangdong, and northern Hainan as core “integrated quality regions”. These findings not only reveal the sensitivity and vulnerability of *A. officinarum* Hance to climate change but also provide spatially explicit guidance for in situ germplasm conservation and the selection of high-quality cultivation bases.

## Simple Summary

This research develops an integrated quality assessment framework for *Alpinia officinarum* Hance, an important medicinal plant in China. By combining multi-model ensemble forecasting of habitat suitability with spatial interpolation of its key bioactive constituent galangin, we project a southwestward shift in suitable habitats under future climate change. The core integrated quality regions were delineated in southeastern Yunnan, southwestern Guangxi, southwestern Guangdong, and northern Hainan. The study provides spatially explicit guidance for the conservation and sustainable cultivation of this species.

## 1. Introduction

*Alpinia officinarum* Hance, a member of the Zingiberaceae family, is a medicinal and edible plant also known as galangal, lesser galangal, or Chinese ginger. Recognized as one of the “Ten Major Guangdong Medicinal Materials” in China [1], it is primarily cultivated in Guangdong, Guangxi, Yunnan, and Hainan provinces. Its earliest documented use can be traced to Ming Yi Bie Lu (Records of Famous Physicians), where it was classified as a medium-grade medicinal substance. In Traditional Chinese Medicine (TCM) theory, *A. officinarum* Hance is characterized by a pungent flavor and warm property, acting on the spleen and stomach meridians. It is commonly employed to warm the stomach, arrest vomiting, dispel cold, and alleviate pain, making it clinically applicable for conditions such as cold and painful epigastric and abdominal regions, belching, acid regurgitation, and vomiting due to stomach cold [2]. As an important traditional herb, *A. officinarum* Hance contains a diverse array of phytochemicals, including diarylheptanoids, volatile oils, flavonoids, glycosides, phenylpropanoids, and various trace elements [3]. Modern pharmacological studies have confirmed that its crude extracts and specific compounds exhibit a broad spectrum of bioactivities, such as antimicrobial, antiviral, antitumor, antioxidant, anti-gastrointestinal hemorrhage, anti-ulcer, and gastric mucosa protective effects [4]. Among these constituents, diarylheptanoids and flavonoids have garnered significant attention from both academia and industry for their potential in antitumor and antioxidant activities, as well as in combating multidrug-resistant bacterial strains [5]. Beyond its medicinal uses, *A. officinarum* Hance is widely utilized in food preservation, essential oil extraction, perfume formulation, culinary seasoning, and health beverage production [6]. Given its remarkable medicinal and edible values, the sustainable development of the *A. officinarum* Hance industry is crucial for meeting market demands and ensuring the quality and safety of the medicinal material.

In addition to its medicinal and culinary values, *A. officinarum* Hance serves as an important agricultural and industrial crop with notable economic significance. Its cultivation is predominantly concentrated in Guangdong Province, especially in Xuwen County, which is renowned as the “home of galangal” and accounts for approximately 90% of the national planting area. However, in recent years, farmer enthusiasm for cultivation has diminished due to its relatively long growth cycle, typically three years, and lower economic returns compared to alternative crops such as sweet potato and banana. Statistical data indicate that the national planting area decreased from about 433.3 hectares in 2007 to roughly 335 hectares in 2011, while the annual yield declined from 7000 to 8000 tons during 2007–2009 to only 3500–4000 tons in 2011. Despite this contraction in production, market demand has remained stable, with an annual consumption of around 6000 tons, primarily as a spice and flavoring agent [7]. In recent years, the price of galangal has shown a steady upward trend, reflecting the dynamics between its supply and demand. This trend underscores the importance of adopting sustainable production strategies to ensure stable market supply and to support the conservation of this valuable resource [8].

*A. officinarum* Hance is a plant of significant medicinal and economic value. Extensive research has established that it is rich in diverse phytochemicals, including diarylheptanoids, flavonoids, volatile oils, and phenylpropanoids, with galangin serving as an important marker compound [9,10,11]. The application of advanced analytical techniques has been crucial in supporting the establishment of reliable quality control standards for this species [12,13]. Pharmacological studies consistently demonstrate a broad spectrum of bioactivities, encompassing anti-inflammatory, antioxidant, antitumor, antibacterial, and gastroprotective effects. Recent evidence also suggests that its polysaccharide components may possess immunomodulatory functions [14,15]. Beyond direct investigations into its chemistry and pharmacology, increasing attention is being paid to the influence of environmental and microbial interactions on its quality. For instance, the composition of endophytic fungi within its rhizomes correlates with plant age, geographic origin, and the content of key metabolites such as volatile oils and galangin, implying a potential role for microbes in regulating biosynthesis [16]. Furthermore, galangal essential oil can positively affect the rhizosphere microbiome of neighboring plants (e.g., *Panax notoginseng*), promoting the growth of beneficial microbial populations and enhancing the host plant’s secondary metabolism [17]. Despite these advances, critical knowledge gaps persist. Existing research is often fragmented, revealing a disconnect between cultivation practices, rhizosphere ecology, and the mechanisms underlying quality formation. This disconnect becomes particularly pronounced in the context of environmental change, as the complex interplay among plant physiology, soil microbial communities, and the accumulation of secondary metabolites remains insufficiently understood [18,19,20,21]. Moreover, a systematic assessment of how this ecologically sensitive species will respond to future climate scenarios—including distribution shifts and adaptive capacity—is still lacking. Therefore, adopting an integrated multi-model approach to simultaneously evaluate its environmental suitability and quality determinants is essential for developing sustainable cultivation and conservation strategies.

A growing body of evidence indicates that climate change poses a severe threat to plant species worldwide, profoundly altering their geographical distributions and habitat suitability [22]. Specifically for *A. officinarum* Hance, this species is highly dependent on warm and humid conditions and is particularly sensitive to low temperatures and seasonal drought. Future climate warming is projected to drive the centroid of its suitable habitat southwestward. Under high-emission scenarios such as SSP585, a pronounced contraction and fragmentation of its suitable habitat are anticipated by the end of the 21st century, highlighting the species’ vulnerability to ongoing climate change [23]. In this context, species distribution models (SDMs) have become essential tools for assessing the impacts of climatic shifts. Among them, the maximum entropy (MaxEnt) model is widely adopted for its reliability with limited occurrence data, while ensemble approaches such as Biomod2 improve predictive robustness by integrating multiple algorithms [16,17,24]. Recent advances further emphasize model parameter optimization; for instance, tools like ENMeval enable the adjustment of regularization multipliers and feature class settings in MaxEnt. Such refinements help mitigate overfitting risks, thereby enhancing the model’s generalizability and ecological interpretability [25,26]. As global climate change intensifies, many rare and endangered species are experiencing rapid contraction or shifts in their suitable ranges. Niche models, which quantify relationships between species and environmental variables, serve as core methodologies for evaluating potential suitable areas, habitat quality, and species-specific climatic responses [27,28,29]. However, predictions from traditional single-model approaches often exhibit considerable uncertainty due to their inherent algorithmic limitations [30].

To address this, the present study adopted a multi-model ensemble strategy for *A. officinarum* Hance [31,32]. This approach integrated a suite of algorithms, including Generalized Linear Models (GLM), Generalized Additive Models (GAM), Gradient Boosting Machines (GBM), Classification Tree Analysis (CTA), Artificial Neural Networks (ANN), Surface Range Envelopes (SRE), Flexible Discriminant Analysis (FDA), Multivariate Adaptive Regression Splines (MARS), Random Forest (RF), and a parameter-optimized Maximum Entropy (MaxEnt) model. By combining validated distribution records of *A. officinarum* Hance with environmental variables filtered through Pearson correlation analysis, we systematically predicted the evolution of its suitable habitats under both current and future climate scenarios. These scenarios encompassed the 2050s and 2090s under four Shared Socioeconomic Pathways (SSP126, SSP245, SSP370, and SSP585) [33,34]. Each model offers distinct advantages: GLM and GAM provide interpretable nonlinear fittings; GBM, RF, and ANN excel at capturing higher-order interaction effects and demonstrate strong predictive performance; CTA, FDA, MARS, and SRE are, respectively, suitable for rule visualization, multi-class discrimination, automated threshold identification, and rapid modeling without requiring missing data handling [35,36]. The MaxEnt model optimized via the ENMeval platform exhibited enhanced robustness under conditions of limited sample size [37]. By integrating these multiple models, this study effectively combined the strengths of individual algorithms, significantly reducing prediction uncertainty. Consequently, it provides a reliable scientific foundation for the conservation of rare species, early warning of biological invasions, and risk assessment under climate change.

In medicinal plant research, relying solely on ecological suitability predictions is no longer sufficient to guide cultivation practices. Recent years have witnessed a paradigm shift in the zoning of Chinese medicinal materials: the spatial distribution information of active ingredients is now incorporated into “quality suitability” analysis. Subsequently, ecological suitability and quality suitability are overlaid to delineate “integrated quality regions” [38]. This approach employs techniques such as kriging interpolation to predict the spatial patterns of constituent contents. Combined with ecological modeling results, it identifies regions capable of simultaneously supporting plant growth and the accumulation of effective compounds, thereby providing direct guidance for “niche-mimicking cultivation” and the planning of high-quality production areas. Based on this framework, the present study collected data on the content of major active ingredients in *A. officinarum* Hance and applied Co-kriging interpolation to generate a spatial map of quality suitability. Ultimately, by performing a weighted overlay of the ecological suitability layer and the quality suitability layer [39,40], a preliminary “integrated quality region” for *A. officinarum* Hance was delineated. This work not only provides a scientific basis for the conservation of galangal germplasm resources but also offers a more comprehensive perspective for planning premium production regions and sustainable management under climate change.

Drawing upon a multi-model ensemble approach, this study was designed to achieve the following interconnected objectives: (1) to systematically identify the dominant environmental factors governing the geographic distribution of *A. officinarum* Hance and quantify their relative contributions; (2) to predict and delineate its potential distribution across China under current climatic conditions; (3) to assess the dynamics of its suitable habitat—including changes in area, spatial pattern evolution, and centroid shift trajectories—under multiple future climate scenarios (SSP126, SSP245, SSP370, and SSP585) for the 2050s and 2090s, while comparing the consistency of predictions across different models; (4) to optimize the parameters of the MaxEnt model, thereby enhancing its predictive performance and ecological interpretability. Furthermore, (5) based on key bioactive constituent data, we employed Kriging spatial interpolation to map the quality suitability distribution of this species. Finally, (6) by integrating the evaluations of ecological suitability and quality suitability for the first time, we constructed an “Integrated Quality Regions” framework for *A. officinarum* Hance. Through these multidimensional analyses, this research aims to comprehensively elucidate the species’ response mechanisms to climate change and the geographic patterns underlying its quality formation. The findings are expected to provide a scientific basis and decision-support for population conservation, habitat restoration, selection of high-quality cultivation regions, and sustainable resource utilization of *A. officinarum* Hance.

## 2. Materials and Methods

### 2.1. Species Data Collection and Processing

**Figure 1:**
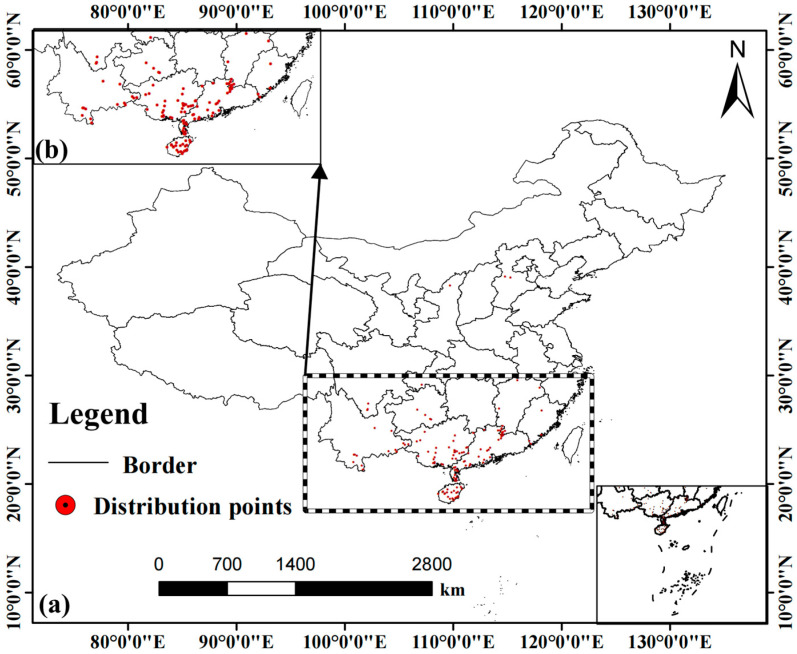
The distribution information of *A. officinarum* Hance: (**a**) Distribution records of *A. officinarum* Hance in China; (**b**) Location of the study area within China.

The geographical distribution data of *A. officinarum* Hance were obtained from three authoritative databases: the Chinese Virtual Herbarium (CVH, http://www.cvh.ac.cn, accessed on 1 December 2025), the National Specimen Information Infrastructure (NSII, http://www.nsii.org.cn/2017/home.php, accessed on 1 December 2025), and the Global Biodiversity Information Facility (GBIF, https://www.gbif.org/, accessed on 3 December 2025). After removing duplicates and verifying geographical coordinates, 149 independent occurrence records with precise latitude and longitude information were initially compiled. To minimize the impact of spatial sampling bias and autocorrelation on model performance, a spatial rarefaction procedure was applied using the “Spatially Rarefy Occurrence Data” tool in the SDM Toolbox, with a filtering distance set to 5 km [41]. This process resulted in 129 high-confidence occurrence records, which were subsequently used for model construction (Figure 1). It should be noted that these data primarily consist of specimen records, which generally do not distinguish between wild and cultivated individuals. Therefore, the modeling dataset may contain both types of occurrences. We did not actively exclude potentially cultivated points, partly because it is difficult to trace the detailed land-use history of each record, and partly because traditional cultivation of *A. officinarum* Hance often resembles semi-wild management within suitable habitats, implying that its fundamental ecological requirements remain consistent with those of wild populations. As the occurrence data may include both wild and cultivated records, the modeling outputs primarily reflect the potential ecological suitability for the species, rather than strictly representing the realized niche of natural populations. This approach aligns with our objective to guide imitation-wild cultivation, thereby providing a basis for selecting sites for “near-natural cultivation”.

To more comprehensively address the inherent collection biases in specimen data (such as the tendency to sample more accessible areas), this study implemented multiple measures during the modeling process. First, during model construction, a large number of pseudo-absence points were randomly selected from the background environment across the entire study area (i.e., China) for comparison. This approach helps to explore the environmental space in unsampled regions, thereby mitigating accessibility bias. Second, a multi-model ensemble strategy was adopted. By integrating various algorithms such as random forest and generalized additive models, differences in their sensitivity to bias were leveraged to smooth potential overfitting from any single model, thus enhancing the robustness of predictions. Finally, strict screening based on environmental variable contribution rates and correlation analysis was applied to ensure that the factors included in the models possess clear ecological relevance rather than merely reflecting biased sampling patterns. Together, these integrated strategies aim to build models on more generalized environmental relationships, thereby providing a more reliable representation of the species’ potential distribution and the spatial patterns underlying its quality formation.

### 2.2. Environmental Variable Selection and Climate Scenario Configuration

To comprehensively characterize the habitat features of *A. officinarum* Hance, this study selected three categories of environmental variables: bioclimatic, soil, and topographic factors. Nineteen bioclimatic variables representing the current period (1970–2000) and future periods were sourced from the WorldClim database (http://www.worldclim.org/, accessed on 20 December 2023) at a spatial resolution of 2.5 arc-minutes. The BCC-CSM2-MR global climate model was adopted as the primary data source for future climate projections due to its demonstrated performance in simulating regional climate patterns across China, particularly daily extreme temperature and precipitation. Within the framework of the Coupled Model Intercomparison Project Phase 6 (CMIP6), four Shared Socioeconomic Pathways (SSPs) were selected to cover a range of radiative forcing scenarios: the sustainable development pathway (SSP126), the intermediate pathway (SSP245), the regional rivalry pathway (SSP370), and the fossil-fueled development pathway (SSP585) [42]. The projection periods were set for the mid-21st century (2050s: 2041–2060) and the late 21st century (2090s: 2081–2100). Soil and topographic data were obtained from the Harmonized World Soil Database (https://www.fao.org/soils-portal/data-hub/soil-maps-and-databases/harmonized-world-soil-database-v12/en/, accessed on 25 December 2023) and the WorldClim database (http://www.worldclim.org/, accessed on 22 December 2023), respectively. All environmental layers were clipped to the extent of China.

To mitigate multicollinearity among environmental variables (Table 1), a two-step filtering procedure was adopted. Firstly, variables with negligible contributions were excluded based on the results of an initial MaxEnt 3.4.4 (AMNH, New York, NY, USA) run. This preliminary screening utilized the built-in jackknife test and variable contribution analysis within MaxEnt to quantify the importance of each variable. Variables exhibiting near-zero contribution rates were considered to have minimal independent explanatory power and were therefore removed. Subsequently, Pearson correlation analysis and variance inflation factor (VIF) calculations were performed on the remaining variable set. For variable pairs showing high correlation (|r| > 0.8), the variable with clearer ecological relevance to *A. officinarum* Hance (based on known physiological traits) and a higher contribution rate in the preliminary model was retained. This practice of screening environmental variables based on contribution and correlation prior to final modeling is a well-established approach for improving model performance and interpretability [43]. The iterative process continued until the VIF values of all selected variables fell below 10. This methodology ensures variable independence while retaining the most ecologically informative predictors, thereby constructing a robust habitat model [44].

### 2.3. Model Construction, Optimization and Evaluation

**Figure 2:**
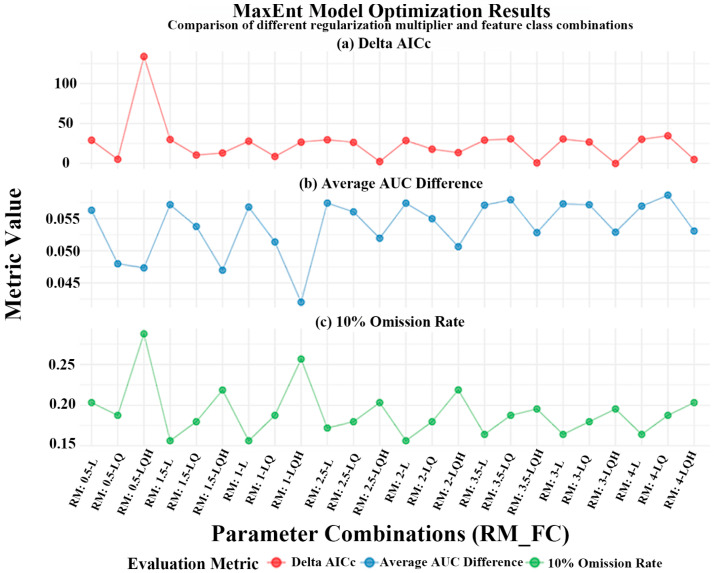
Optimization of the MaxEnt model: (**a**) Delta AICc; (**b**) Average AUC Difference; (**c**) 10% Omission Rate.

To simulate the potential distribution of *A. officinarum* Hance, we utilized MaxEnt software version 4.4.1. Both the model optimization and subsequent analyses were conducted based on the subset of key environmental variables selected through the screening procedure detailed in Section 2.2. To enhance the model’s generalization ability and mitigate overfitting risks, critical parameters of the MaxEnt model were optimized using the ENMeval package in RStudio 4.4.1 (PBC, Boston, MA, USA). This process involved systematic adjustments to the regularization multiplier and feature classes. The regularization multiplier was tested across a range from 0.5 to 4.0 in increments of 0.5, while the evaluated feature classes included L, LQ, H, LQH, LQHP, and LQHPT. The optimal parameter combination was selected by minimizing the corrected Akaike Information Criterion [45,46]. Consequently, this parameter tuning was performed on the pre-screened, non-redundant variable set, with results presented in Figure 2. The fundamental model settings were configured as follows: 75% of the species occurrence records were randomly assigned as the training set, with the remaining 25% reserved for testing. The maximum number of iterations was set to 1,000,000. This high upper limit is a common precautionary practice in ecological modeling to ensure sufficient opportunity for convergence; in actual runs, the process is controlled by a convergence threshold (default 1.0 × 10^−5^), which prevents prediction instability due to premature termination. A 10-fold cross-validation was applied, and the jackknife test was used to assess the contribution of individual environmental variables. Model performance was evaluated using the area under the receiver operating characteristic curve (AUC). An AUC value ≥ 0.8 was considered indicative of reliable predictions, while values between 0.9 and 1.0 denote high predictive accuracy [47]. Using the optimized model, we predicted the potentially suitable habitats under current conditions and for future periods (2050s and 2090s) across four Shared Socioeconomic Pathways: SSP126, SSP245, SSP370, and SSP585.

In all suitability distribution maps and subsequent spatial classifications of the integrated quality index, this study consistently applied the Natural Breaks method. This approach optimizes inter-class variance, enabling objective classification based on the inherent distribution characteristics of each raster dataset, thereby avoiding the subjective bias that may arise from manually setting uniform thresholds. As a result, the delineation of categories such as “high-suitability regions” accurately reflects the natural structure of the data within each specific scenario, enhancing the ecological interpretability of classification outcomes for individual spatiotemporal settings. Maintaining methodological consistency across scenarios is crucial for cross-scenario comparison. Although the absolute ranges of suitability probabilities differ among various climate scenarios, applying the same classification algorithm allows the comparison to focus on spatial dynamics—such as centroid shifts and changes in suitable area—of “relatively high-suitability regions” identified by a unified standard. This ensures that the observed trends are driven by environmental changes due to climate variation rather than by technical discrepancies arising from inconsistent classification methods. Thus, this approach provides a methodological foundation for reliable comparisons across periods and emission pathways in this study [48].

To comprehensively evaluate prediction uncertainty and enhance the robustness of the results, this study further constructed a multi-model ensemble framework. Initially, nine algorithms were considered, including Generalized Linear Model (GLM), Generalized Additive Model (GAM), Gradient Boosting Machine (GBM), Classification Tree Analysis (CTA), Artificial Neural Network (ANN), Surface Range Envelope (SRE), Flexible Discriminant Analysis (FDA), Multivariate Adaptive Regression Splines (MARS), and Random Forest (RF). Among these, FDA was excluded from actual modeling due to failure in convergence, while the remaining eight algorithms were successfully executed. These eight algorithms were implemented using the Biomod2 package in R Studio 4.4.1 [49]. Each algorithm was run with 10 replicates, applying a data split of 75% for training and 25% for validation. Additionally, 1000 pseudo-absence points were generated to improve the model’s discriminative capability.

Model performance was comprehensively evaluated using the True Skill Statistic (TSS), the Kappa coefficient, and the Area Under the Curve (AUC). The selection criteria were set as follows: AUC ≥ 0.80, TSS ≥ 0.60, and Kappa ≥ 0.50 [50,51]. The final ensemble prediction was generated through a two-step procedure. First, individual models run within the Biomod2 framework that met all three performance thresholds were selected. Then, the predictions from these selected Biomod2 models were combined with the prediction from the independently optimized MaxEnt model described earlier in this section to produce a consensus ensemble prediction. Only models satisfying the aforementioned thresholds were incorporated into the final ensemble. The resulting probability raster from the ensemble was subsequently reclassified into unsuitable, generally suitable, moderately suitable, and highly suitable areas using the natural breaks method. By comparing changes in the suitable area and spatial patterns across different scenarios and time periods, this study systematically elucidates the potential impacts of climate change on the distribution of *A. officinarum* Hance.

### 2.4. Spatial Interpolation Analysis of Galangin Content

To clarify the geographical variation in the quality of *A. officinarum* Hance, galangin—a marker compound specified in the 2025 edition of the Chinese Pharmacopoeia—was selected as the chemical indicator for quality assessment [52,53]. Through a systematic search of literature published between 2008 and 2019 in the China National Knowledge Infrastructure (https://www.cnki.net) and Google Scholar (https://scholar.google.com) (search date: 24 November 2025), a total of 30 records with precise geographical coordinates and galangin content determined by standardized methods such as high-performance liquid chromatography were collected (Supplementary Table S1). Although the sample size is limited, the data cover the core genuine producing areas of *A. officinarum* Hance, which sufficiently reflects quality differences across major environmental gradients. To identify key environmental drivers for the spatial interpolation of galangin content, Spearman’s rank correlation analysis was performed between the collected galangin content values and an initial set of 37 environmental variables. This non-parametric method was chosen because it does not assume linear relationships or normal distribution of the data, making it suitable for exploring potential monotonic relationships between secondary metabolite content and environmental factors. Based on this dataset, a spatial distribution model of galangin content was constructed using Co-kriging interpolation in ArcGIS 10.8 (Esri, Redlands, CA, USA) [54]. This approach visually presents the geographical variation pattern of galangin content and provides a data foundation for analyzing the coupled “environment–quality” relationship.

### 2.5. Delineation of Integrated Quality Regions

To identify production potential areas that combine high ecological suitability with superior medicinal quality, an integrated quality evaluation system based on “ecological–chemical” synergy was constructed. This system integrates two key types of information: firstly, an ecological suitability probability raster derived from multi-model ensemble predictions, which characterizes the species’ survival potential; and secondly, a galangin content raster generated through Co-kriging interpolation, representing the chemical quality of the medicinal material. The integrated quality index was calculated using a weighted overlay method. For the baseline scenario, equal weights (0.5 each) were assigned to ecological suitability and chemical quality suitability. This weighting scheme is not arbitrary; rather, it is grounded in the rationale that, within the framework of “ecological–chemical” coordinated zoning for medicinal plants, both survival potential and medicinal value are regarded as equally fundamental dimensions for defining “geo-authenticity” (*daodi*) and achieving sustainable resource use. In the absence of precise experimental data to quantify their relative contributions, assigning equal weights represents a transparent, neutral, and widely adopted strategy in related studies [55]. To test the robustness of the key conclusions under this baseline scheme, sensitivity analyses were conducted with alternative weighting scenarios that emphasized either ecological (0.7:0.3) or chemical (0.3:0.7) aspects (see Section 3.6 and Supplementary Figure S3). Finally, the integrated quality index obtained under the baseline weights was classified using the natural breaks method, thereby delineating integrated quality regions at different levels. This approach enables the precise identification of key areas where ecological and chemical attributes are optimally synergistic [38], providing a scientific basis for defining the geo-authenticity of *A. officinarum* Hance and for guiding its cultivation planning.

### 2.6. Centroid Migration Analysis

Using the “Mean Center” tool within the Spatial Statistics module of ArcGIS 10.8 [56,57], we calculated the geometric centroid of the suitable habitat for *A. officinarum* Hance for each time period (current, 2050s, 2090s) under each climate scenario. This analysis aimed to quantify the directional shift and displacement distance of the suitable area in geographic space, thereby revealing the response trend and spatial migration pattern of *A. officinarum* Hance to future climate change.

## 3. Results

### 3.1. Model Parameter Optimization and Performance Evaluation

The parameter optimization of the model was conducted using the key environmental variable set selected through the procedure detailed in Section 2.2. Compared with the default parameter settings (RM = 1, FC = Auto features), the systematically tuned MaxEnt model (RM = 3, FC = LQH) demonstrated superior overall performance. The optimized model achieved a Delta AICc of 0.00, indicating an optimal balance between model complexity and goodness-of-fit. Although the mean AUC difference increased slightly (0.0529), the mean validation AUC improved to 0.9422, while both the standard deviation of AUC difference (0.0155) and the 10% test omission rate (0.1953) decreased significantly, reflecting enhanced discriminative ability and stability (Figure 2). Furthermore, the tuned parameter combination effectively mitigated overfitting risks, substantially reducing the number of model coefficients (from approximately 60 to 19) and narrowing the gap between training and test gain from 0.216 to 0.001, which notably improved the model’s generalizability and explanatory power. Therefore, this parameter set was selected for subsequent prediction of suitable habitats.

The predictive performance of the eight candidate algorithms was evaluated and compared (Supplementary Figure S1, Table 2). Among them, the RF, GAM, GBM, and MARS models demonstrated superior performance, with AUC, TSS, and Kappa values all exceeding 0.90, 0.60, and 0.60, respectively. These four algorithms met all predefined performance thresholds (AUC ≥ 0.80, TSS ≥ 0.60, Kappa ≥ 0.50) and were therefore selected for subsequent ensemble modeling. This outcome indicates their effectiveness in capturing the distribution patterns of *A. officinarum* Hance, confirming their suitability for simulating habitat suitability in this study.

### 3.2. Identification of Dominant Environmental Factors and Analysis of Suitable Thresholds

Through an initial screening of variable contributions based on the MaxEnt model, combined with Pearson correlation analysis and variance inflation factor (VIF) testing, this study selected 12 key environmental factors from an initial set of 37 for final model construction (Supplementary Figure S2). These include critical climatic variables such as Bio1 (annual mean temperature), Bio4 (temperature seasonality), and Bio17 (precipitation of the driest quarter), with detailed variable information provided in Table 1. The optimized MaxEnt model demonstrated excellent statistical performance, achieving a test AUC of 0.949 and a minimal training-test gain difference of approximately 0.001. This indicates an effective balance between predictive accuracy and the risk of overfitting, ensuring the reliability of the results. To quantify the relative importance of these key environmental drivers, the percentage contribution of each variable in the optimized MaxEnt model (with RM = 3 and FC = LQH) was analyzed, as presented in Table 3. Although the final distribution prediction employed a multi-model ensemble (Biomod2) to enhance robustness, the MaxEnt model was specifically chosen for variable importance analysis. This is because it provides clear and interpretable estimates of individual variable contributions, a feature particularly suited for identifying dominant ecological limiting factors.

**Figure 3:**
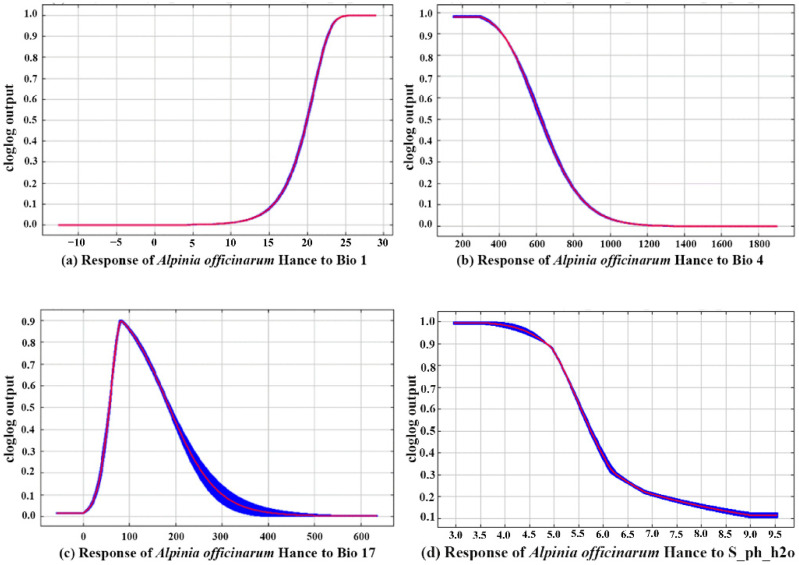
The response curve of dominant environmental factors: (**a**) Bio1 Annual mean temperature; (**b**) Bio4 Temperature seasonality; (**c**) Bio17 Precipitation of the driest quarter; (**d**) S_ph_h2o Subsoil pH.

Response curve analysis (Figure 3) unveiled nonlinear associations between *A. officinarum* Hance and its core environmental determinants, alongside their respective suitability thresholds (presence probability > 0.5). As summarized in Table 3, the four leading variables (Bio1, Bio4, Bio17, and S_ph_h2o) collectively accounted for over 98% of the model contribution, whereas each of the remaining factors (e.g., T_clay, Elev, T_silt) contributed less than 1%. Accordingly, a detailed threshold examination was conducted specifically for these four dominant climatic factors, given their primary role in constraining the species’ distribution. Table 4 illustrates that the growth of this species is strongly reliant on warm and humid climatic regimes. Specifically, the suitable annual mean temperature (Bio1) spans from 19.96 to 29.05 °C, with an optimal range of 25.63–29.05 °C. Precipitation during the driest quarter (Bio17) must be maintained between 56.64 and 185.65 mm, and values exceeding 83.69 mm are considered more favorable. Temperature seasonality (Bio4) exhibits a suitable range of 149.92 to 622.96, reflecting the species’ pronounced sensitivity to seasonal thermal variation. In addition, the appropriate subsoil pH ranges from 2.95 to 5.74, with an optimal band of 2.95–3.49. Other edaphic and topographic attributes, including topsoil clay content, elevation, and topsoil silt content, also exert auxiliary influences on habitat suitability. Collectively, the potential distribution of *A. officinarum* Hance is primarily governed by the dual stresses of low temperature and seasonal aridity. Its core distribution areas demonstrate a strong spatial correspondence with subtropical humid monsoon climates, which are characterized by warm and moist conditions, ample precipitation, and adequate moisture availability during the dry season.

### 3.3. Potential Distribution Pattern Under Current Climatic Conditions

**Figure 4:**
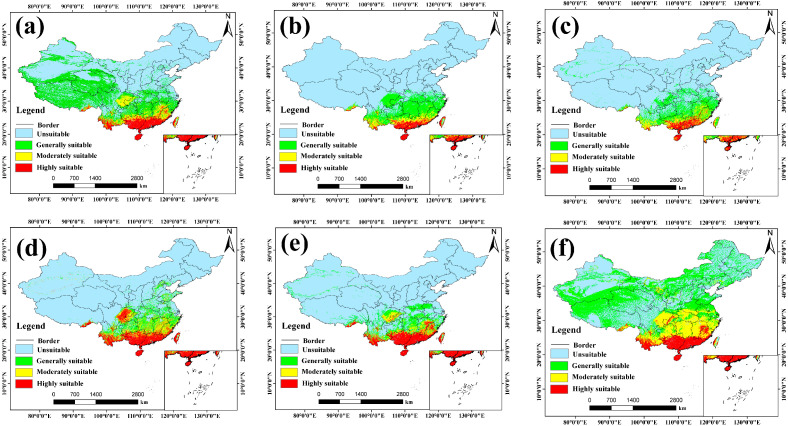
Potential distribution of *A. officinarum* Hance under present climatic conditions predicted by different modeling approaches. (**a**) Ensemble model, (**b**) MaxEnt, (**c**) RF, (**d**) GAM, (**e**) GBM, and (**f**) MARS.

Predictions from the ensemble model reveal the potential spatial distribution pattern of *A. officinarum* Hance under current climatic conditions (Figure 4). Overall, the suitable habitats for this species exhibit a pronounced latitudinal zonation, being primarily confined to regions south of the Yangtze River Basin. This reflects a strong ecological preference for the warm and humid climates characteristic of subtropical and tropical regions.

The model evaluation metrics (TSS = 0.86, AUC = 0.97) indicate that the ensemble model possesses exceptionally high predictive accuracy and robustness. Spatially, the highly and moderately suitable areas constitute the core ecological niche of *A. officinarum* Hance, concentrated predominantly in South China (Guangdong, Guangxi, Hainan, Fujian) and Southwest China (southern Yunnan, the fringes of the Sichuan Basin, and Guizhou). The ample hydrothermal conditions in these regions provide optimal habitats for its growth. In contrast, the generally suitable areas extend northward to the middle-lower reaches of the Yangtze River and the Qinling-Huaihe line, forming the peripheral region of the species’ distribution. It is noteworthy that while the total suitable area covers approximately 38% of China’s terrestrial landmass, the truly high-quality core habitat (highly suitable area) accounts for only 4.84% of the national area, implying a high selectivity of this species for specific microenvironments.

Regarding model comparison, the spatial patterns predicted by four individual models: MaxEnt, GAM, GBM, and RF, closely aligned with the ensemble model, all accurately identifying the humid southern provinces as the primary suitable areas (see specific values in Supplementary Table S2). However, the predictions from the MARS model displayed significant over-generalization. Its projected range anomalously expanded into arid and alpine regions such as Xinjiang and Inner Mongolia, leading to a substantial overestimation of the suitable area to over half of the country. This deviation likely stems from the MARS algorithm’s overfitting of response curves for certain environmental factors, failing to accurately capture ecological thresholds under extreme climatic conditions. Consequently, the ensemble model, by smoothing out the predictive biases of individual models, yielded a more conservative and ecologically plausible distribution prediction, making it a more appropriate baseline for subsequent analyses.

### 3.4. Projected Potential Distribution Under Future Climate Scenarios

**Figure 5:**
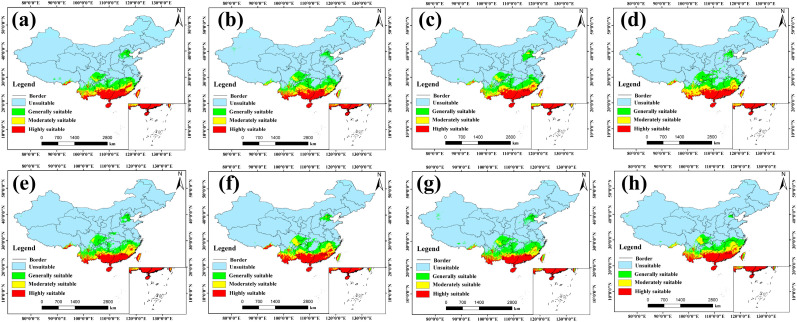
Potential distribution of *A. officinarum* Hance under future climate conditions as modelled by Ensemble model: (**a**) 2041–2060, SSP126; (**b**) 2081–2100, SSP126; (**c**) 2041–2060, SSP245; (**d**) 2081–2100, SSP245; (**e**) 2041–2060, SSP370; (**f**) 2081–2100, SSP370; (**g**) 2041–2060, SSP585; (**h**) 2081–2100, SSP585.

Ensemble projections derived from five species distribution models revealed complex responses of the potential suitable habitat of *A. officinarum* Hance to future climate change. Although the underlying algorithms differed across models, with the exception of MARS, the remaining models (MaxEnt, RF, GAM, GBM) exhibited broadly convergent spatiotemporal patterns under the various Shared Socioeconomic Pathways (see Supplementary Figures S3–S6). The ensemble model further consolidated the predictions from these robust models and yielded a coherent distribution pattern (Figure 5).

Model results consistently indicate that greenhouse gas emission intensity will markedly alter the suitable habitat patterns of *A. officinarum* Hance. Under the low-emission scenario (SSP126), this species exhibits strong adaptive resilience. Most models suggest that although the area of suitable habitat may fluctuate slightly in the 2050s, the total suitable area tends to stabilize or even recover modestly by the 2090s, as corroborated by predictions from the MaxEnt (Supplementary Figure S3a,b), RF (Supplementary Figure S4a,b), and ensemble models (Figure 5a,b). This trend implies that *A. officinarum* Hance is capable of adapting to moderate climatic changes.

**Figure 6:**
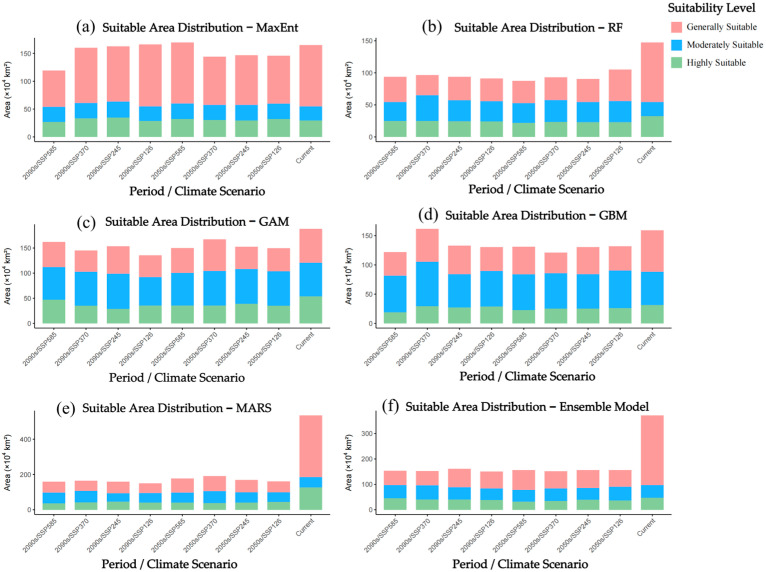
Suitable area of *A. officinarum* Hance under different future climate scenarios and periods: (**a**) MaxEnt; (**b**) RF; (**c**) GAM; (**d**) GBM; (**e**) MARS; (**f**) Ensemble model.

In contrast, under the high-emission scenario (SSP585), this species is projected to face substantial challenges. While certain models such as GAM (Supplementary Figure S5g) suggest a transient expansion of suitable habitats during the early 2050s, a pronounced contraction is anticipated by the 2090s as the effects of sustained greenhouse forcing accumulate. This trajectory is consistently corroborated by predictions from MaxEnt (Supplementary Figure S3h), RF (Supplementary Figure S4h), GBM (Supplementary Figure S6h), and the ensemble model (Figure 5h). Notably, the highly suitable areas under SSP585 exhibit evident fragmentation, implying that extreme climatic conditions may have surpassed the physiological tolerance thresholds of the species, thereby imposing long-term constraints on its distribution. The projected changes in the extent of suitable habitats across different models are summarized in Figure 6, which provides a direct comparison of relative area shifts across scenarios and time periods, thereby further quantifying the patterns described above.

From a spatial perspective, the centroid of the suitable habitat for *A. officinarum* Hance exhibits a clear pattern of dynamic migration. Projections from the ensemble model indicate that during the near-term future (2050s), highly suitable areas remain concentrated in traditional core regions, including southern Yunnan, Guangxi, Guangdong, Hainan, southern Fujian, and western Taiwan (Figure 5a,c,e,g). Under future climate scenarios, the core distribution continues to be centered in South China, particularly in Guangdong, Guangxi, and southern Fujian, highlighting the critical role of these regions as climatic refugia. However, notable shifts are observed at the margins of the distribution. Under the SSP126 scenario, moderately suitable areas exhibit a northward expansion into southern Jiangxi, Zhejiang, and even southern Hubei, as illustrated by the MaxEnt (Supplementary Figure S3a,b) and GAM (Supplementary Figure S5a,b) models—a trend consistent with the general ecological expectation of poleward range shifts under global warming. In contrast, under the high-emission SSP585 scenario, intensified thermal stress and altered precipitation patterns along the southeastern coast lead to a westward retreat and concentration of highly suitable habitat toward inland areas such as eastern Sichuan and southwestern Chongqing, accompanied by a decline in suitability in traditionally core regions like southwestern Guangdong.

Notably, the predictions generated by the MARS model diverged substantially from those of the other four individual models and the ensemble model (Supplementary Figure S7). MARS projected an exceptionally broad suitable habitat for both current and future periods, encompassing most of northern China—a pattern inconsistent with the range contraction trends toward the 2090s indicated by the other models. Given that *A. officinarum* Hance is a typical tropical/subtropical medicinal plant, the MARS outputs likely overestimate its fundamental niche breadth beyond its actual physiological tolerance limits. Consequently, when formulating conservation strategies, caution should be exercised regarding the extreme values predicted by MARS; greater reliance should instead be placed on the more robust projections derived from MaxEnt, RF, and the ensemble model.

In summary, future climate change is anticipated to exert profound impacts on the geographical distribution of *A. officinarum* Hance. Aggressive emissions reduction pathways (SSP126) are conducive to maintaining long-term population stability, whereas high-emission scenarios (SSP585) may lead to substantial loss and degradation of core habitats. The consensus projection of the ensemble model further reinforces this conclusion and provides more reliable spatially explicit evidence to support regional conservation planning and climate adaptation strategies.

### 3.5. Spatiotemporal Dynamics of Centroid Shift in Suitable Habitats

**Figure 7:**
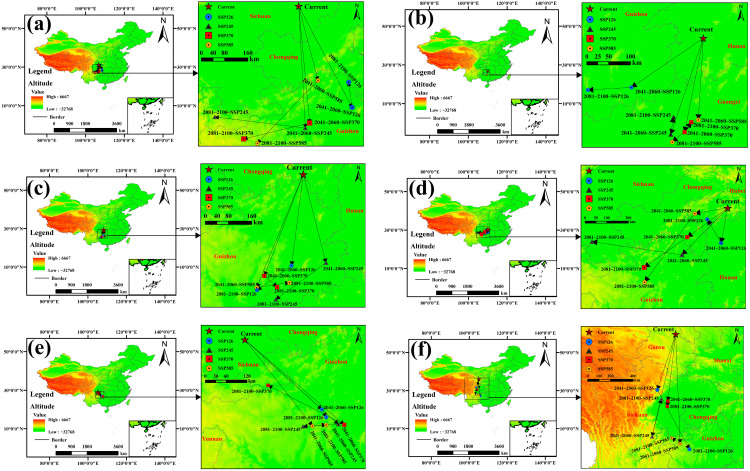
Centroid migration trajectories of the total suitable area for *A. officinarum* Hance under future climate scenarios: (**a**) Ensemble model; (**b**) MaxEnt; (**c**) RF; (**d**) GAM; (**e**) GBM; (**f**) MARS.

Simulations of the centroid of the suitable habitat for *A. officinarum* Hance under future climate scenarios, based on five modeling approaches, reveal a pronounced southwestward migration trend despite variations in the specific trajectories and distances projected by each model (Figure 7). This directional shift suggests that, in response to climate warming, the species tends to seek new climatic refugia in southwestern inland regions characterized by higher elevations or lower latitudes, such as the Yunnan–Guizhou Plateau and adjacent areas.

Specifically, the MaxEnt, RF, and GAM models consistently indicate a sustained southwestward retreat of the centroid from its current location near the Hunan-Hubei-Chongqing border toward regions such as Guizhou and Guangxi. Among these, the RF model projects the most dramatic displacement under the high-emission SSP585 scenario, with a migration distance reaching 417.76 km by the 2050s, implying that extreme climate change may accelerate the reshaping of the species’ distribution pattern. In contrast, although the GBM model exhibits fluctuations toward the southeast, its final centroid remains in the eastern part of the Yunnan–Guizhou Plateau, which does not contradict the overall southwestward contraction trend. Notably, the MARS model predicts an exceptionally long southeastward migration (>500 km), which may entail a risk of overfitting given that its initial centroid (located in Gansu) falls far outside the actual ecological amplitude of the species; therefore, this result should be interpreted with caution.

The centroid migration trajectory derived from the ensemble model further consolidates and reinforces the general trend observed among the robust individual models (Figure 7a). The current centroid of the ensemble prediction is located at approximately 106.82° E, 31.17° N, and under future climate scenarios, it exhibits a consistent and systematic shift toward the southwest. By the 2050s, the migration distances across SSP scenarios range from 263.17 km (SSP585) to 422.02 km (SSP245). This displacement continues into the 2090s, with distances varying between 87.59 km (SSP126) and 284.66 km (SSP585). These findings indicate that the ensemble model not only aligns with the directional consensus of most single-model simulations, but also captures a clear gradient in migration magnitude corresponding to the intensity of radiative forcing. This further supports the interpretation that southwestward migration constitutes a primary spatial strategy for this species in response to climate change.

Overall, the southwestward migration of the habitat centroid is more pronounced under the SSP585 scenario than under SSP126, indicating that stronger climate forcing may compel the species to undergo more substantial geographic displacement to maintain its ecological niche. This trend is consistently reinforced by the consensus projections derived from the ensemble model, further highlighting the strategic importance of southwestern China as a core area for future germplasm resource conservation.

### 3.6. Delineation of Integrated Quality Regions

Spearman correlation analysis identified topsoil silt content (T_silt) and subsoil pH (S_ph_h2o) as key positive drivers of galangin accumulation among the 37 environmental variables assessed (Supplementary Figure S8). The significant positive correlations between these factors and galangin content suggest that specific soil physical structure and pH conditions promote the synthesis of this secondary metabolite.

**Figure 8:**
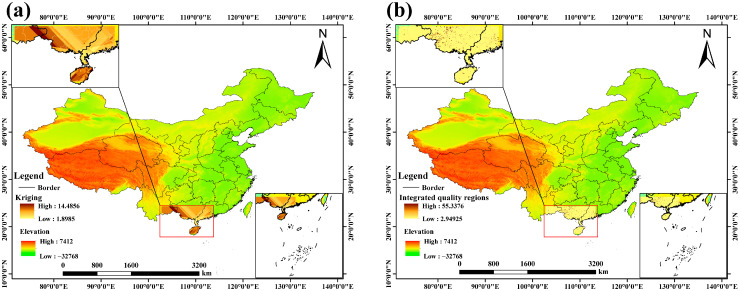
Integrated quality regions evaluation. (**a**) Co-kriging analysis of galangin; (**b**) Integrated quality regions of *A. officinarum* Hance.

Spatial heterogeneity of galangin was further revealed by Co-Kriging interpolation based on these key drivers (T_silt and S_ph_h2o). Regions with high galangin content showed clear spatial aggregation, primarily encompassing southeastern Yunnan, southwestern Guangxi, southern Guangdong, and the entirety of Hainan (Figure 8a). This distribution pattern not only confirms the regulatory role of soil texture and pH in quality formation but also provides chemogeographic evidence for identifying superior germplasm resources.

By integrating the chemical quality suitability index with the ecological suitability index through an equal-weight overlay approach—each assigned a weight of 0.5, a common practice in current medicinal plant ecological–chemical zoning studies intended to equally prioritize species survival potential and medicinal value—we constructed an integrated quality evaluation model for *A. officinarum* Hance (Figure 8b). The resulting zoning map revealed that southeastern Yunnan, southwestern Guangxi, southwestern Guangdong, and northern Hainan constitute the regions of highest integrated quality. Subsequent weight sensitivity analysis (Supplementary Figure S9) confirmed that the spatial configuration of these core high-quality areas remained largely unchanged even when the ecological-to-chemical weight ratio was varied (e.g., 0.7:0.3 or 0.3:0.7), demonstrating the robustness of our delineation. These areas achieve spatial synergy between suitable ecological niches and high metabolite-accumulation potential, thereby forming the central regions for the conservation and sustainable utilization of high-quality *A. officinarum* Hance resources. Collectively, this model transcends the limitations of single-dimension environmental assessments and establishes a scientific paradigm for production layout under the dual constraints of ecological suitability and chemical quality.

## 4. Discussion

In contrast to the recent predictive approach by Kang et al., which primarily relied on an optimized MaxEnt model [58], the present study developed an integrated forecasting framework that incorporates 129 distribution records and five modeling algorithms—MaxEnt, RF, GAM, GBM, and MARS. This multi-model ensemble substantially enhances the robustness of projections under future climate uncertainty. Under the SSP585 scenario, for instance, individual models such as MaxEnt and RF predicted a pronounced contraction of suitable habitats by the 2090s (approximately 27.62~36.21%), whereas GBM and MARS suggested relatively stable or even locally expanding trends. Such marked divergence in predictions not only highlights the limitations of single-algorithm approaches in capturing the complexity of species responses to environmental change [59], but also affirms the critical role of ensemble modeling in reducing predictive bias and improving the reliability of conclusions [60].

Multi-model analysis identified precipitation of the driest quarter (Bio17) as a key factor constraining the distribution of *A. officinarum* Hance, with its lower suitability threshold (ca. 83.69 mm) quantifying the species’ high sensitivity to seasonal drought. This statistical inference aligns closely with physiological experiments on ginger (*Zingiber officinale*), a related species within the same family. Liu et al. (2024) demonstrated that drought stress induces stomatal closure in ginger to maintain water status and photosynthetic capacity, indicating stomatal behavior as a crucial drought-response mechanism [61]. By analogy, it is plausible that *A. officinarum* Hance relies on similar stomatal regulation to cope with dry seasons in its natural habitat. The 83.69 mm precipitation threshold thus represents not merely a statistical boundary, but an “ecological critical point” essential for sustaining basic physiological functions. This mechanistic insight explains why the species is predominantly distributed in the warm-humid monsoon regions of southeastern China, where dry-season precipitation remains sufficient, and also implies its potential vulnerability under future drying trends. Furthermore, the inclusion of microenvironmental factors such as soil texture underscores that habitat selection in *A. officinarum* Hance is governed by the dual regulation of macroclimatic conditions and local edaphic properties.

Building upon yet moving beyond previous studies focused primarily on ecological suitability prediction, this work breaks new ground by incorporating the spatial heterogeneity of galangin content into the evaluation framework, marking a paradigm shift towards assessing “high-quality production suitability” [54]. Utilizing the Co-Kriging interpolation technique within the ArcGIS platform, we mapped the spatial distribution of galangin content across major production areas in China. This analysis revealed a pronounced enrichment pattern, with significantly higher concentrations clustered in southeastern Yunnan, Guangxi, southwestern Guangdong, and Hainan. By constructing and overlaying an “ecology-quality” comprehensive index, we clearly delineated these regions as the “integrated quality core regions” for *A. officinarum* Hance. Sensitivity analysis under alternative weighting schemes confirmed that the spatial pattern of these core integrated quality regions remained stable (Supplementary Figure S9). This zoning outcome simultaneously considers ecological suitability and the potential for secondary metabolite accumulation, providing direct spatial decision-making support for identifying genuine producing areas, screening superior germplasm, and planning cultivation regions. It strongly promotes the evolution of medicinal plant zoning from a simplistic “can it grow?” approach to a more sophisticated “can it produce high quality?” paradigm.

Building on previous efforts in ecological suitability prediction, this study further advances the field by incorporating the spatial heterogeneity of galangin content into the evaluation framework—a methodological innovation that signals a paradigm shift toward assessing “high-quality production suitability” [62]. Using Co-kriging interpolation within the ArcGIS platform, we generated a spatial distribution map of galangin content across the main producing regions of China. The results revealed a distinct pattern of enrichment in southeastern Yunnan, southwestern Guangxi, southwestern Guangdong, and northern Hainan. By constructing and overlaying an ecological–quality composite index, we explicitly delineated these areas as the “integrated quality core regions” for *A. officinarum* Hance. This zoning outcome, which simultaneously considers both ecological suitability and secondary metabolite accumulation potential, offers direct spatial decision support for the identification of genuine producing areas, the selection of elite germplasm, and the planning of cultivation regions. Collectively, this work substantially advances the paradigm of medicinal plant zoning, shifting its focus from merely assessing “whether a species can grow” to determining “whether it can be cultivated with high quality”.

It should be noted that the spatial interpolation of galangin content was based on a relatively limited sample size (*n* = 30); therefore, the integrated quality zones should be considered a regional-scale, first-order approximation rather than precise, site-specific production guidelines. Future studies with expanded phytochemical sampling across broader geographic ranges would enable more refined delineation of quality zones and better capture local-scale variability in secondary metabolite accumulation.

In this integrated assessment, we adopted two key methodological assumptions and provided justification for each. First, regarding the temporal projection of chemical quality, the spatial pattern of galangin content suitability was generated based on current data and assumed to remain stable under future climate scenarios. This simplification is grounded in the identification of edaphic properties—specifically topsoil silt content (T_silt) and subsoil pH (S_ph_h2o)—as the dominant environmental factors regulating the accumulation of this compound. Unlike climatic variables, these soil attributes are controlled by parent material and long-term pedogenic processes, and their properties change only slowly over the timescales considered in this study. Consequently, the quality production potential determined by the soil matrix is unlikely to be directly or rapidly altered by climate change [63,64]. Although this assumption inevitably overlooks the complexity of plant physiological and biochemical responses to changing climatic conditions, it represents a necessary simplification in the absence of process-based models capable of projecting dynamic shifts in secondary metabolites. Nonetheless, it offers a pragmatic and operational basis for identifying areas where future climate suitability coincides with inherently favorable soil conditions [65]. Second, for the construction of the integrated quality index, we applied an equal-weighting scheme between the ecological and chemical dimensions. This baseline choice aligns with the prevailing paradigm in current medicinal plant “ecological–chemical” synergistic zoning studies, and is intended to assign equal importance to the two core objectives: species survival potential and medicinal value. More importantly, systematic sensitivity analyses comparing alternative weighting schemes (e.g., 0.7:0.3, 0.5:0.5, and 0.3:0.7) revealed that the spatial pattern of the delineated core quality regions remained highly stable and largely insensitive to weight adjustments. This finding confirms a strong spatial coupling between ecological suitability and chemical quality suitability. Therefore, even if future research enables more precise calibration of the weights, the core regions identified under the current equal-weight baseline—such as southeastern Yunnan and southwestern Guangxi—retain reliable reference value and provide robust spatial guidance for geoheritage authentication and conservation-oriented cultivation planning.

Simulations based on the BCC-CSM2-MR climate scenarios indicate divergent futures. Under the low-emission SSP126 pathway, the total suitable habitat for *A. officinarum* Hance remains stable or even shows slight expansion by the end of the century, bene-fiting from optimized hydrothermal conditions in some regions. In stark contrast, under the high-emission SSP585 scenario, particularly by the 2090s, both the highly suitable and total suitable areas are projected to contract significantly. This negative response to persistent warming and altered precipitation patterns lends strong support to the “large genome constraint hypothesis,” which posits that high temperatures and drought re-strict the distribution of species with large genomes [66]. Notably, the future centroid of suitable habitat shows a tendency to migrate towards mid-elevation areas in the southwest, especially the eastern Yunnan–Guizhou Plateau, with the migration magnitude increasing under stronger emission scenarios. While this trajectory differs from the northwestward shift reported for some karst plants by Kang et al., it aligns with the common ecological preference for humid and shaded environments within the Zingiberaceae family. This suggests that under climatic stress, *A. officinarum* Hance tends to retreat to mid-altitude refugia with more balanced hydrothermal conditions.

The realization of potential suitable habitats is governed not only by climatic factors but also by multiple constraints including species dispersal capacity, interspecific interactions, topographic barriers, and anthropogenic disturbances [67]. Given that *A. officinarum* Hance predominantly reproduces via rhizomes, its natural dispersal ability is relatively weak. This limitation, combined with ongoing harvesting pressure and habitat fragmentation confronting wild populations, suggests that actual migration rates may lag behind the pace of climatic suitability shifts. Furthermore, treating chemical quality as a static attribute represents a methodological limitation, since the complex physiological and biochemical responses of plants to elevated CO_2_, altered temperatures, and changing hydrological regimes could ultimately affect galangin biosynthesis. Although this study has certain shortcomings—including limited spatial coverage of phytochemical samples and incomplete coupling with non-climatic factors such as land use and pest pressure—the established ecological–chemical synergy framework nonetheless provides an operational tool for adaptive management. We therefore recommend prioritizing in situ conservation and ex situ breeding programs in future potential high-suitability areas (e.g., the eastern Yunnan–Guizhou Plateau), while simultaneously reinforcing habitat restoration within current distribution ranges. Integrating remote-sensing-based dynamic monitoring with population genomics would facilitate more comprehensive, multi-scale risk assessments, thereby enhancing both the scientific basis and resilience of conservation and sustainable resource utilization strategies for this species.

## 5. Conclusions

This study innovatively integrates multi-model ensemble forecasting with a dual evaluation system encompassing both ecological and chemical dimensions, systematically elucidating the spatiotemporal dynamics of *A. officinarum* Hance and its quality formation mechanisms under climate change. The findings reveal that *A. officinarum* Hance, as a typical warm-humid subtropical ecotype, has its geographical distribution co-limited by annual mean temperature (19.96~29.05 °C), temperature seasonality (149.92~622.96 °C), and precipitation of the driest quarter (56.64~185.65 mm), demonstrating high sensitivity to both low-temperature and drought stress. Currently, the suitable habitats are primarily concentrated in Yunnan, Guangxi, Guangdong, Fujian, and Hainan, covering a total area of approximately 367.83 × 10^4^ km^2^. However, under different future climate scenarios, significant response variations emerge: habitats remain relatively stable under the low-emission scenario (SSP126), whereas under medium- to high-emission scenarios (SSP370/585), the suitable areas face substantial contraction and fragmentation risks. Notably, the centroid of its suitable habitat exhibited a clear southwestward shift, particularly toward mid-elevation areas in the eastern Yunnan–Guizhou Plateau, reflecting an adaptive strategy to avoid thermal stress. This southwestward migration trend was consistently supported by the ensemble analysis of centroid shifts, reinforcing its recognition as a consensus spatial response of this species to climate change.

Furthermore, by integrating spatial heterogeneity information of the key bioactive compound galangin, this study established an “ecological–quality” comprehensive evaluation index, which precisely delineated southeastern Yunnan, southwestern Guangxi, southwestern Guangdong, and northern Hainan as core regions for high-quality production. This provides spatial guidance for the paradigm shift from habitat suitability assessment to “high-quality and suitable production” planning.

It should be noted that the conclusions of this study are drawn on certain methodological premises and datasets, and thus entail corresponding uncertainties. First, the spatial pattern of galangin content was derived from current data and assumed to remain stable under future climate scenarios. Although this simplification is based on the relative stability of the dominant edaphic drivers, it does not fully capture the complex physiological and biochemical responses of plants to climate change. Second, the equal-weight synthesis of the integrated quality index serves as a baseline weighting scheme; its validity is supported by sensitivity analyses confirming the spatial robustness of the core production regions. Nevertheless, more precise weight calibration awaits further support from ecophysiological research. Additionally, the projections rely on output from a single global climate model (BCC-CSM2-MR), and the species occurrence data may contain sampling biases, while the spatial coverage of phytochemical samples also requires expansion. Future work could enhance the completeness and accuracy of predictions by incorporating multiple climate models, increasing sample sizes, and coupling species dispersal mechanisms with dynamic models of quality trait responses. Despite these limitations, the ecological–chemical synergy framework developed in this study, together with the identified core regions, still provides critical scientific evidence and decision support for in situ conservation, site selection of artificial cultivation bases, and adaptive management of *A. officinarum* Hance resources.

## Figures and Tables

**Table 1:** All environmental variables.

Variables	Name	Unit
Bio1	Annual mean temperature	°C
Bio2	Mean diurnal temperature range	°C
Bio3	Isothermality	/
Bio4	Temperature seasonality	/
Bio5	Maximum temperature of the warmest month	°C
Bio6	Minimum temperature of the coldest month	°C
Bio7	Temperature annual range	°C
Bio8	Mean temperature of the wettest quarter	°C
Bio9	Mean temperature of the driest quarter	°C
Bio10	Mean temperature of the warmest quarter	°C
Bio11	Mean temperature of the coldest quarter	°C
Bio12	Annual precipitation	mm
Bio13	Precipitation of the wettest month	mm
Bio14	Precipitation of the driest month	mm
Bio15	Precipitation seasonality	/
Bio16	Precipitation of the wettest quarter	mm
Bio17	Precipitation of the driest quarter	mm
Bio18	Precipitation of the warmest quarter	mm
Bio19	Precipitation of the coldest quarter	mm
Awc_class	Soil available water content	%
Slope	Slope	◦
Elev	Elevation	m
Aspect	Aspect	/
T_ph_h2o	Topsoil pH	-log(H+)
S_ph_h2o	Subsoil pH	-log(H+)
T_oc	Topsoil organic carbon content	%weight
S_oc	Subsoil organic carbon content	%weight
T_clay	Topsoil clay content	%weight
S_clay	Subsoil clay content	%weight
T_sand	Topsoil sand content	%weight
S_sand	Subsoil sand content	%weight
T_silt	Topsoil silt content	%weight
S_silt	Subsoil silt content	%weight
T_ece	Topsoil electrical conductivity	ds/m
S_ece	Subsoil electrical conductivity	ds/m
T_caco3	Topsoil carbonate or lime content	%weight
S_caco3	Subsoil carbonate or lime content	%weight

**Table 2:** AUC, TSS, and Kappa values for predicting the potential geographic distribution of *A. officinarum* Hance using multiple models including RF, GAM, GBM and MARS.

Indicator	Algorithm							
	RF	GAM	GBM	MARS	CTA	GLM	ANN	SRE
AUC	0.954	0.94	0.939	0.93	0.887	0.854	0.783	0.65
TSS	0.615	0.737	0.787	0.761	0.78	0.659	0.564	0.3
Kappa	0.627	0.62	0.653	0.636	0.593	0.514	0.473	0.363

**Table 3:** Importance of key environmental factors affecting the distribution of *A. officinarum* Hance in China.

Variables	Name	Percent Contribution (%)
Bio1	Annual mean temperature	71.2
Bio4	Temperature seasonality	13.8
Bio17	Precipitation of the driest quarter	12.1
S_ph_h2o	Subsoil pH	1.3
T_clay	Topsoil clay content	0.6
Elev	Elevation	0.5
T_silt	Topsoil silt content	0.3
Aspect	Aspect	0.1

**Table 4:** The suitable range for the dominant environmental factors.

Variable	Suitable Range	Adaptive Threshold
Bio1	19.96~29.05 °C	25.63~29.05 °C
Bio4	149.92~622.96 °C	149.92~295.34 °C
Bio17	56.64~185.65 mm	83.69 mm
S_ph_h2o	2.95~5.74	2.95~3.49

## Data Availability

Data can be made available on reasonable request.

## Supplementary Materials

The following supporting information can be downloaded at: https://www.mdpi.com/article/10.3390/biology15040369/s1, Figure S1: Accuracy verification results for eight single-model algorithms. Figure S2: Correlation Heatmap of Environmental Factors. Figure S3: Potential distribution of *A. officinarum* Hance under future climate conditions as modelled by MaxEnt. Figure S4: Potential distribution of *A. officinarum* Hance under future climate conditions as modelled by RF. Figure S5: Potential distribution of *A. officinarum* Hance under future climate conditions as modelled by GAM. Figure S6: Potential distribution of *A. officinarum* Hance under future climate conditions as modelled by GBM. Figure S7: Potential distribution of *A. officinarum* Hance under future climate conditions as modelled by MARS. Figure S8: Results of Spearman’s correlation analysis for component-relatedness. Figure S9: Integrated Quality Regions of *A. officinarum* Hance under different weighting schemes. Table S1: Composition data of active components in *A. officinarum* Hance from different geographical origins. Table S2: Suitable area of *A. officinarum* Hance under different future climate scenarios and periods.
