# Supplementary material for: Zoning of Integrated Quality Regions for Alpinia officinarum Hance Based on a Multi-Model Evaluation System
Source: Biology (Basel). 2026 Feb 22;15(4):369. doi: 10.3390/biology15040369 (PMC12937820; doi:10.3390/biology15040369)
Supplement: Supplementary file 1 [file biology-15-00369-s001.zip › biology-4123412-supplementary.pdf]

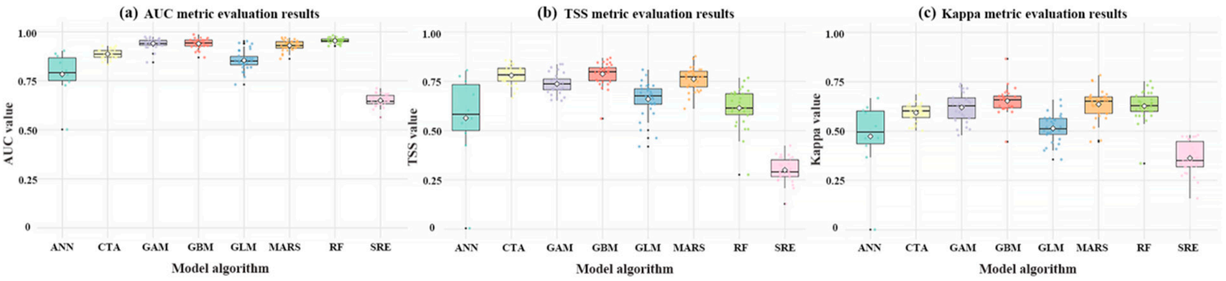

Supplementary Figure S1. Accuracy verification results for eight single-model algorithms. (a) AUC metric evaluation results; (b) TSS metric evaluation results; (c) Kappa metric evaluation results.

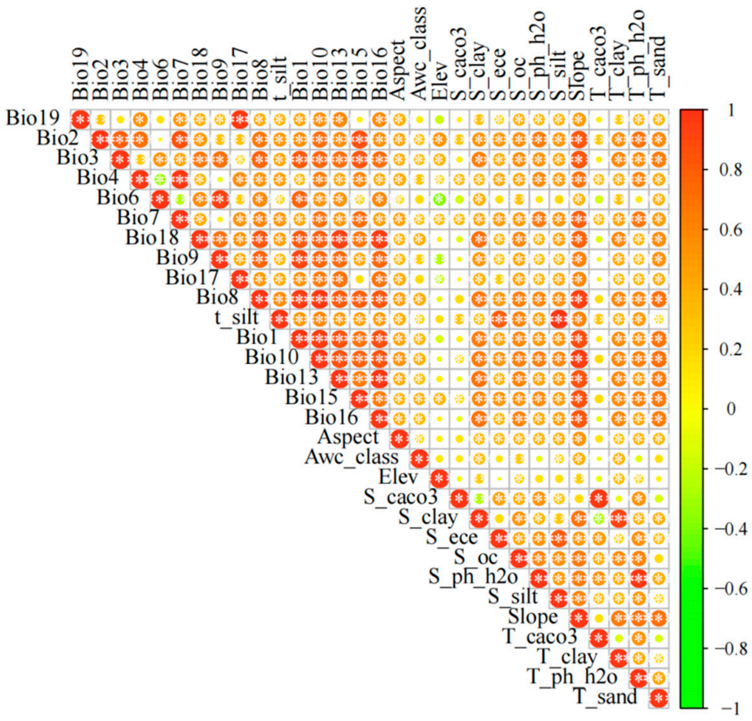

Supplementary Figure S2. Correlation Heatmap of Environmental Factors.

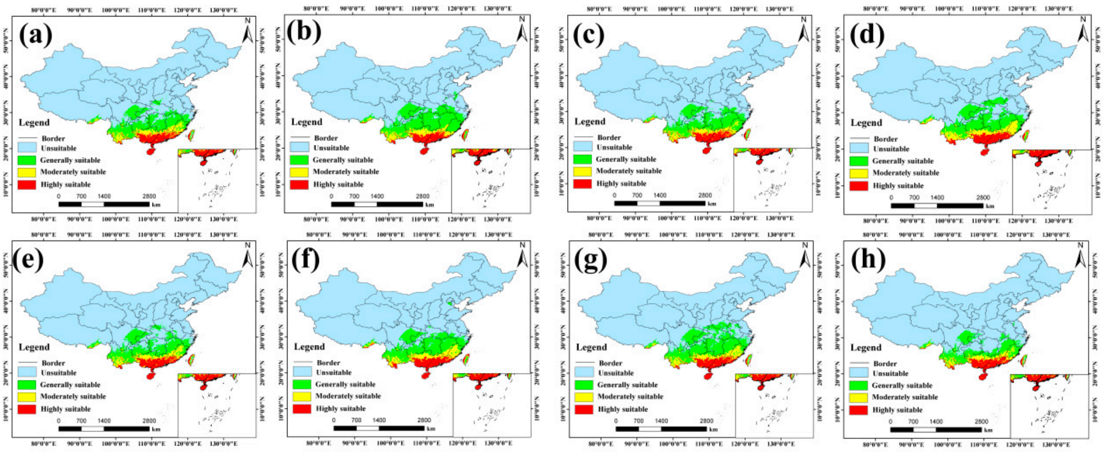

Supplementary Figure S3. Potential distribution of *A. officinarum* Hance under future climate conditions as modelled by MaxEnt: (a) 2041–2060, SSP126; (b)2081–2100, SSP126; (c) 2041–2060, SSP245; (d) 2081–2100, SSP245; (e) 2041–2060, SSP370; (f) 2081–2100, SSP370; (g) 2041–2060, SSP585; (h) 2081–2100, SSP585.

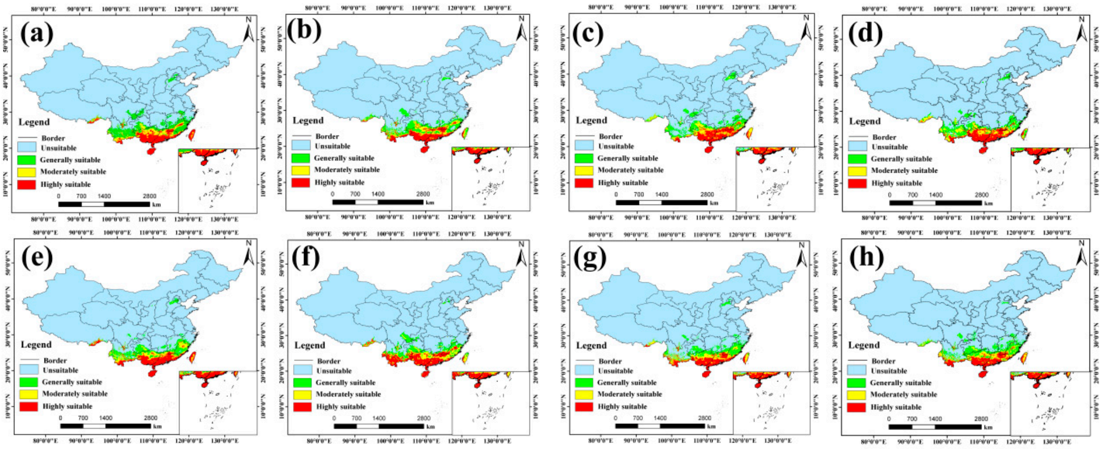

Supplementary Figure S4. Potential distribution of *A. officinarum* Hance under future climate conditions as modelled byRF: (a) 2041–2060, SSP126; (b)2081–2100, SSP126; (c) 2041–2060, SSP245; (d) 2081–2100, SSP245; (e) 2041–2060, SSP370; (f) 2081–2100, SSP370; (g) 2041–2060, SSP585; (h) 2081–2100, SSP585.

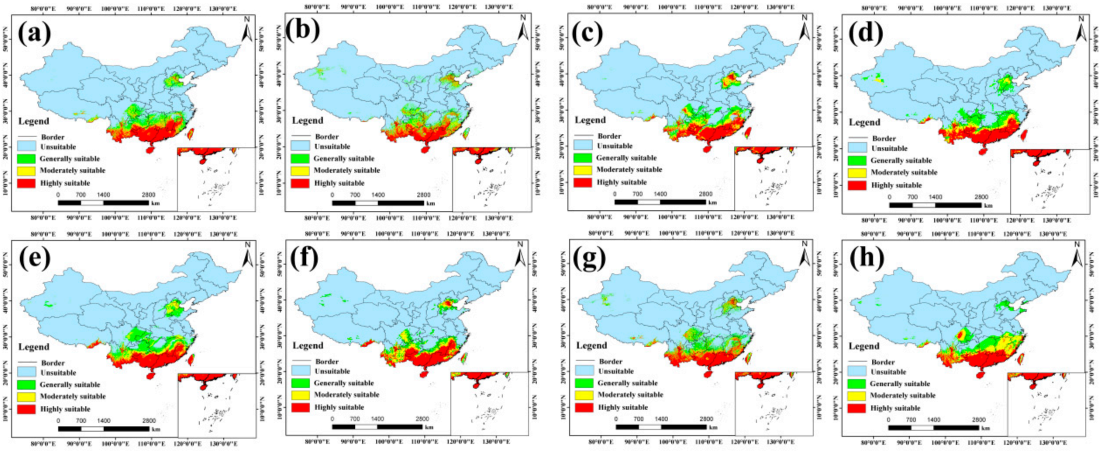

Supplementary Figure S5. Potential distribution of *A. officinarum* Hance under future climate conditions as modelled by GAM: (a) 2041–2060, SSP126; (b)2081–2100, SSP126; (c) 2041–2060, SSP245; (d) 2081–2100, SSP245; (e) 2041–2060, SSP370; (f) 2081–2100, SSP370; (g) 2041–2060, SSP585; (h) 2081–2100, SSP585.

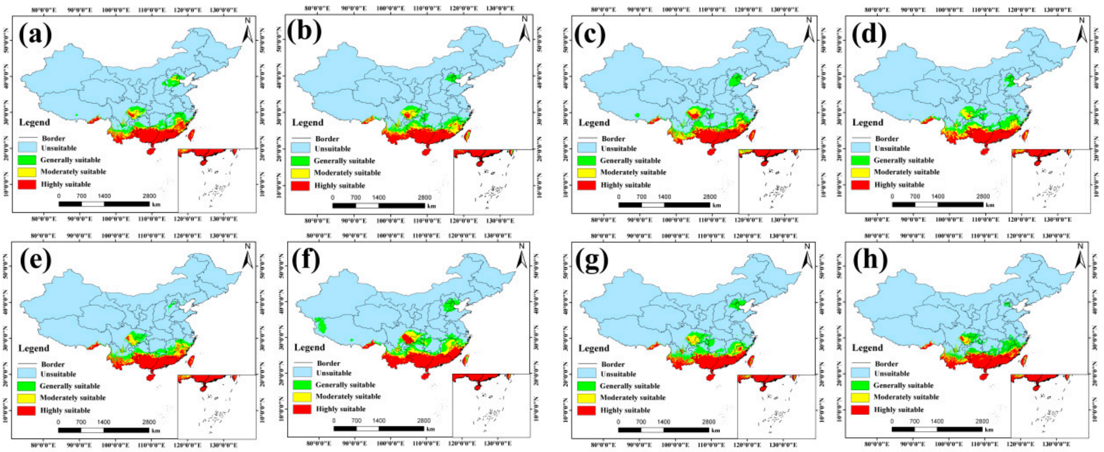

Supplementary Figure S6. Potential distribution of *A. officinarum* Hance under future climate conditions as modelled by GBM: (a) 2041–2060, SSP126; (b)2081–2100, SSP126; (c) 2041–2060, SSP245; (d) 2081–2100, SSP245; (e) 2041–2060, SSP370; (f) 2081–2100, SSP370; (g) 2041–2060, SSP585; (h) 2081–2100, SSP585.

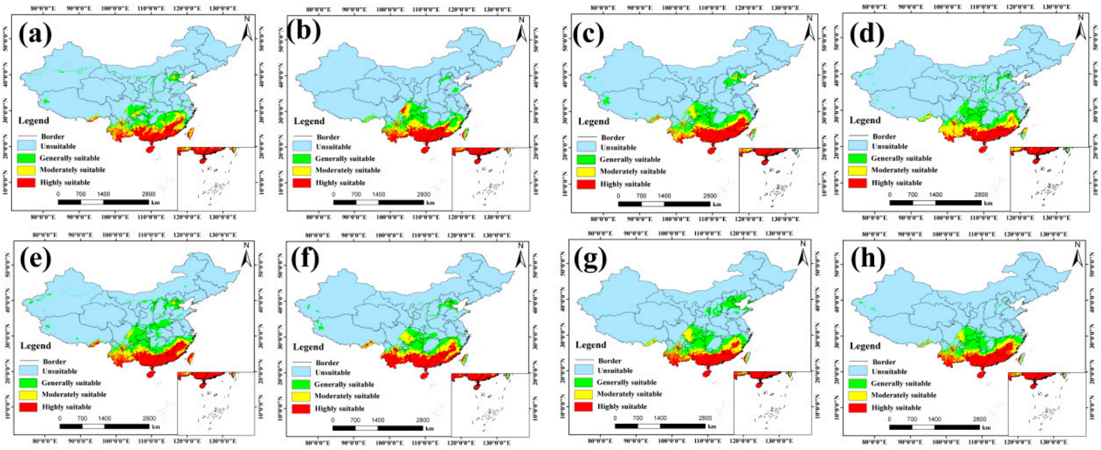

Supplementary Figure S7. Potential distribution of *A. officinarum* Hance under future climate conditions as modelled by MARS: (a) 2041–2060, SSP126; (b)2081–2100, SSP126; (c) 2041–2060, SSP245; (d) 2081–2100, SSP245; (e) 2041–2060, SSP370; (f) 2081–2100, SSP370; (g) 2041–2060, SSP585; (h) 2081–2100, SSP585.

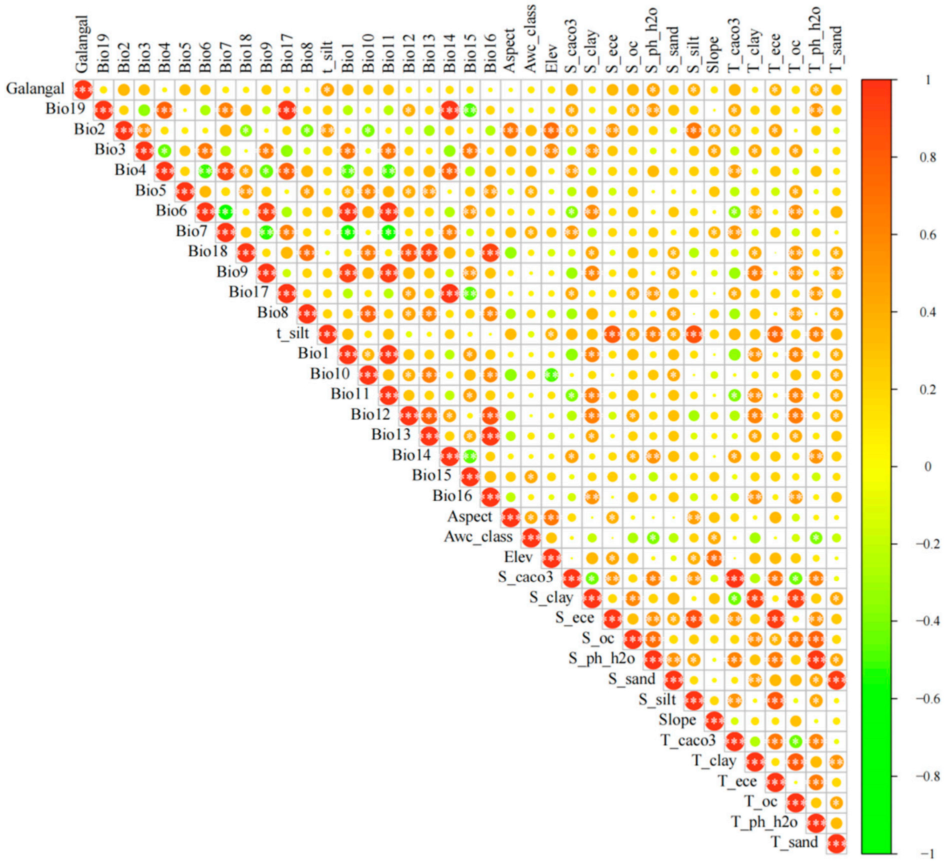

Supplementary Figure S8. Results of Spearman's correlation analysis for component-relatedness.

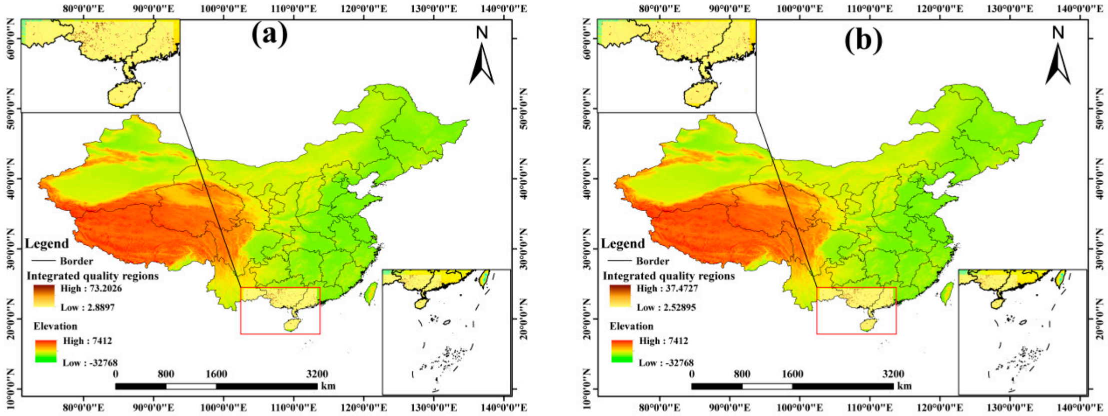

Supplementary Figure S9. Integrated Quality Regions of *A. officinarum* Hance under different weighting schemes: (a) Ecological : Chemical = 0.7 : 0.3; (b) Ecological : Chemical = 0.3 : 0.7.

Supplementary Table S1. Composition data of active components in *A. oficinarum* Hance from different geographical origins.

Sample	Geographic Origin	Latitude (°N)	Longitude (°E)	Galangin Content (mg/g)	Collection Time
S1	Xuwen County, Zhanjiang City, Guangdong Province, China	20.336002	110.338237	6.4	2014
S2	Haikang County, Guangdong Province, China	23.099728	113.295691	7.09	1996
S3	Jiangchuan County, Yuxi City, Yunnan Province, China	24.305879	102.754541	8.37	1998
S4	Xuwen County, Zhanjiang City, Guangdong Province, China	20.340164	110.31913	10.2425	2014
S5	Wenchang City, Hainan Province, China	22.51877	110. 103411	12.01935	2013
S6	Haikou City, Hainan Province, China	19.681232	110.565222	7.6517	2013
S7	Haikou City, Hainan Province, China	19.565669	110.627499	10.2915	2013
S8	Ding'an County, Hainan Province, China	19.478408	110.453133	6.0623	2013
S9	Lingao County, Hainan Province, China	19.601838	109.734129	6.7195	2013
S10	Jiangmen City, Guangdong Province, China	22.598786	113. 154439	8.24	2014
S11	Bobai County, Yulin City, Guangxi Zhuang Autonomous Region, China	22.279214	109.982409	7. 117	2014
S12	Xuwen County, Zhanjiang City, Guangdong Province, China	20.332193	110. 182422	8.4	2014
S13	Xuwen County, Zhanjiang City, Guangdong Province, China	20.32127443	110.3321442	10.9	2013
S14	Xuwen County, Zhanjiang City, Guangdong Province, China	20.42577786	110.4670123	11.8	2013
S15	Xuwen County, Zhanjiang City, Guangdong Province, China	20.48888245	110.3335213	11.4	2013
S16	Lingao County, Hainan Province, China	19.91960184	109.697327	13.3	2013
S17	Lingshui County, Hainan Province, China	18.51306155	110.0439604	14.8	2013
S18	Suixi County, Zhanjiang City, Guangdong Province, China	21.38465329	110.3282944	6.69	2019
S19	Leizhou City, Guangdong Province, China	20.79970057	110.3281669	6.49	2019
S20	Xuwen County, Zhanjiang City, Guangdong Province, China	20.37019446	110.2967289	8.22	2019
S21	Xuwen County, Zhanjiang City, Guangdong Province, China	20.32526719	110.2987337	5.89	2019
S22	Xuwen County, Zhanjiang City, Guangdong Province, China	20.33871519	110.2916239	6.66	2019
S23	Xuwen County, Zhanjiang City, Guangdong Province, China	20.31775381	110.3190627	7.31	2019
S24	Leizhou City, Guangdong Province, China	20.64116856	110.4606354	6.26	2019
S25	Xuwen County, Zhanjiang City, Guangdong Province, China	20.33023516	110.2456602	7. 11	2019

Supplementary Table S1 (Continued). Composition data of active components in *A. oficinarum* Hance from different geographical origins.

Sample	Geographic Origin	Latitude (°N)	Longitude (°E)	Galangin Content (mg/g)	Collection Time
S26	Xuwen County, Zhanjiang City, Guangdong Province, China	20.34147678	110.2636913	10.88	2019
S27	Xuwen County, Zhanjiang City, Guangdong Province, China	20.37108104	110.2344888	5.46	2019
S28	Xuwen County, Zhanjiang City, Guangdong Province, China	20.41827399	110.4506712	7.36	2019
S29	Leizhou City, Guangdong Province, China	20.72567927	109.792495	5.7	2019
S30	Luchuan County, Yulin City, Guangxi Zhuang Autonomous Region, China	22.32778621	110.2705765	7.512	2008

Supplementary Table S2. Suitable area of *A. oficinarum* Hance under different future climate scenarios and periods.

Model	Periods	Climate scenarios	Generally suitable area (× 10^4 km^2)	Moderately suitable area (× 10^4 km^2)	Highly suitable area (× 10^4 km^2)	Total suitable area (× 10^4 km^2)
MaxEnt	Current	—	109.76	29.63	25.36	164.75
	2050s	SSP126	86.05	32.20	27.42	145.66
		SSP245	89.51	29.43	27.99	146.93
		SSP370	86.40	30.35	27. 18	143.93
		SSP585	109.26	32.35	27.98	169.59
	2090s	SSP126	111.34	28.71	26.09	166. 14
		SSP245	99.28	34.49	29.05	162.82
		SSP370	98.94	33.39	27.81	160. 14
		SSP585	65.23	26.79	27.23	119.25
RF	Current	—	92.85	32.36	22.07	147.28
	2050s	SSP126	49.28	22.80	33. 15	105.24
		SSP245	35.85	22.95	31.55	90.34
		SSP370	35.60	23.49	34.03	93. 11
		SSP585	34.63	21.83	30.79	87.25
	2090s	SSP126	35.61	23.91	31.70	91.22
		SSP245	36.33	24.60	32.78	93.71
		SSP370	31.06	25.06	40.23	96.35
		SSP585	39.46	24.72	29.78	93.96
GAM	Current	—	67. 18	54. 13	66.63	187.94
	2050s	SSP126	45.65	35.30	68.61	149.56
		SSP245	43.92	39. 16	68.97	152.05
		SSP370	63.44	35.60	68.51	167.55
		SSP585	49.79	35.39	64.75	149.93
	2090s	SSP126	43.60	35.51	56.49	135.61
		SSP245	54.39	29. 15	69.87	153.42
		SSP370	42.22	35.26	67.64	145. 12
		SSP585	50.42	46.95	64.68	162.05
GBM	Current	—	70.70	31.51	57.01	159.23
	2050s	SSP126	41.01	26.05	64.48	131.53
		SSP245	46.23	25.32	59.07	130.62
		SSP370	35.29	25. 14	60.53	120.96
		SSP585	47.23	22.43	61.25	130.91
	2090s	SSP126	41.00	28.85	60.82	130.66
		SSP245	49.01	26.97	57. 13	133. 11
		SSP370	56.45	29. 11	76.01	161.57
		SSP585	40.02	19.08	62.69	121.79

Supplementary Table S2 (Continued). Suitable area of *A. oficinarum* Hance under different future climate scenarios and periods.

Model	Periods	Climate scenarios	Generally suitable area (× 10^4 km^2)	Moderately suitable area (× 10^4 km^2)	Highly suitable area (× 10^4 km^2)	Total suitable area (× 10^4 km^2)
MARS	Current	—	349.25	126.73	58.93	534.90
	2050s	SSP126	61.93	45.01	54.00	160.94
		SSP245	70.91	40.24	58.22	169.37
		SSP370	85.08	37.83	67.57	190.48
		SSP585	79.62	40.74	56.22	176.59
	2090s	SSP126	54.95	40.44	54.51	149.91
		SSP245	66.08	46.20	47.03	159.31
		SSP370	57.88	41.84	65.27	164.99
		SSP585	61.96	37.03	60.67	159.65
Ensemble Model	Current	—	273.88	47.40	50.23	371.51
	2050s	SSP126	65.55	36.51	54.00	156.06
		SSP245	70.44	39.94	45.94	156.32
		SSP370	67.18	34.97	49.37	151.52
		SSP585	77.16	32.20	46.46	155.82
	2090s	SSP126	66.89	38.98	45.07	150.95
		SSP245	72.24	40.85	47.97	161.06
		SSP370	56.06	40.47	56.15	152.68
		SSP585	55.79	45.77	51.85	153.42
